# High-throughput UV-photofragmentation studies of thymine and guanine†

**DOI:** 10.1039/d3cp00328k

**Published:** 2023-04-17

**Authors:** Siwen Wang, Yerbolat Dauletyarov, Peter Krüger, Daniel A. Horke

**Affiliations:** a Institute for Molecules and Materials, Radboud University, Heijendaalseweg 135 6525 AJ Nijmegen The Netherlands d.horke@science.ru.nl

## Abstract

High-throughput photofragmentation studies of thymine and guanine were performed at 257 and 343 nm and for a wide range of ionisation laser intensities. Combining a continuous laser-based thermal desorption source with femtosecond multiphoton ionisation using a 50 kHz repetition rate laser allowed us to produce detailed 2D maps of fragmentation as a function of incident laser intensity. The fragmentation was distinctly soft, the parent ions being at least an order of magnitude more abundant than fragment ions. For thymine there was a single dominant fragmentation channel, which involves consecutive HNCO and CO losses. In contrast, for guanine there were several competing ones, the most probable channel corresponding to CH_2_N_2_ loss through opening of the pyrimidine ring. The dependence of parent ion abundance on the ionisation laser intensity showed that at 257 nm the ionisation of thymine is a 1 + 1 resonance enhanced process through its open-shell singlet state.

## Abstract 2

High-throughput photofragmentation studies of thymine and guanine were performed at 257 nm and 343 nm and for a wide range of ionisation laser intensities.

## Graphical abstract

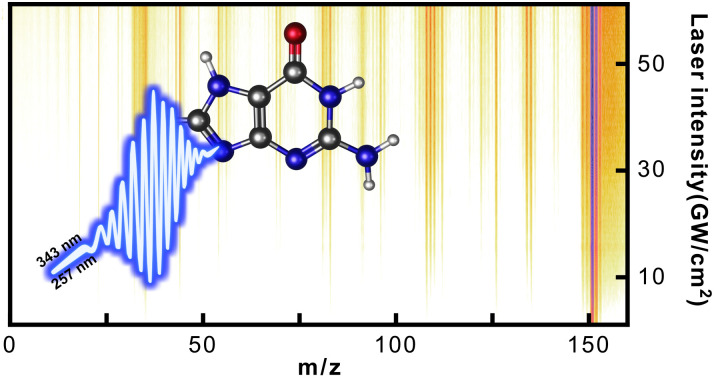

## 1. Introduction

The fragmentation of DNA bases received considerable attention, both experimental and theoretical, over the last half-century, starting with the pioneering electron-impact studies by Rice and coauthors^1,2^ in the mid-1960s, not long after the discovery of DNA itself in the early 1950s.^3^ In particular, the fragmentation mass spectra of DNA bases were obtained employing various ionisation methods based on photon-,^4–7^ electron-,^1,2,8–14^ proton-,^15,16^ and ion-impact,^8,17–19^ as well as collisions with neutral species.^20^

In the last decades this research was mainly driven by the desire of the community to shed more light onto the physical and physico-chemical stages of DNA damage, such as base modifications, single and double strand-breaks, induced by UV-photons either directly or through the production of slow secondary electrons, and consequently reactive oxygen species.^21–23^ Besides its scientific merits, understanding the process of DNA damage is an essential prerequisite for improving the effectiveness of the current cancer radiation therapy techniques.^24^

This paper complements the extensive existing research on the fragmentation of DNA bases by combining a soft vaporisation technique, laser-based thermal desorption (LBTD), with femtosecond multiphoton ionisation (fs-MPI). LBTD is related to an older technique, laser-induced acoustic desorption,^25,26^ but uses a continuous desorption laser instead of a pulsed one.^27^ Hence the desorption mechanism in LBTD cannot be impulse-driven, but is considered purely thermal. It is a unique technique that allows efficient and soft desorption of a sample into the gas phase as intact neutral molecules,^28–30^ in contrast to conventional desorption/ionisation techniques for non-volatiles such as matrix-assisted laser desorption ionisation,^31^ electrospray ionisation,^32^ or desorption electrospray ionisation^33^ which produce ions. LBTD is also significantly ‘softer’ than conventional laser desorption involving direct irradiation of the sample matrix,^34^ which can often lead to significant fragmentation and contamination of the molecular sample.^35^ LBTD is thus especially relevant when working with non-volatile or thermally unstable compounds, as has been demonstrated, for example, by Poully and coworkers, who successfully desorbed intact nucleosides.^27^ Furthermore, by decoupling sample desorption from ionisation, LBTD offers a great control over the latter process.

In this paper, we report fragmentation mass spectra of thymine and guanine following fs-MPI as a function of ionisation laser intensity at wavelengths of 257 nm and 343 nm. While thymine has previously been desorbed using LBTD,^27^ this is the first report of successfully vaporising the non-volatile guanine molecule using this approach. We obtained that, at 257 nm, thymine cations are produced by means of 1 + 1 resonance enhanced multiphoton ionisation (REMPI) through its open-shell singlet state. Meanwhile, at 343 nm, thymine cations are produced by means of MPI without resonance enhancement. Likewise, for guanine, at both wavelengths, the ionisation process does not involve any intermediate resonance state, and fs-MPI is a generally applicable and very soft ionisation approach. Utilizing a high repetition rate femtosecond laser system allowed us to conduct these studies at very high throughput, such that the precise intensity dependence of the fragmentation and ionisation processes could be captured in a short time and we report the first appearance intensities and photon orders for multiphoton ionisation/fragmentation of guanine. The 2D fragmentation mass spectra collected in this fashion yield additional insight into the underlying fragmentation pathways and can furthermore be considered as unique ‘fingerprints’ of a particular molecular structure.

## 2. Experimental methods

**Fig. 1:**
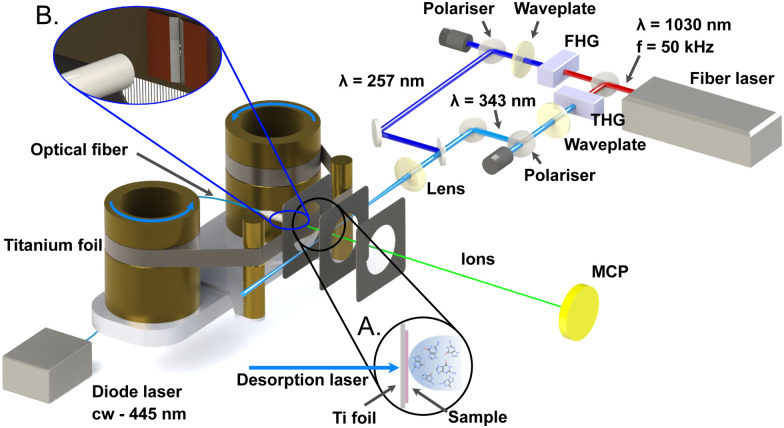
Schematic of the experimental setup. The LBTD source consists of two cylinders that move the foil with the deposited sample, constantly providing fresh sample. Desorption is driven by the continuous output of a fiber-coupled laser diode. The resulting plume of molecules is ionised by the third or fourth harmonic of a high repetition-rate femtosecond fiber laser, and the intensity controlled by a half-wave plate and polariser. Produced ions are analysed using a custom time-of-flight mass spectrometer. For further details see the text.

A detailed description of our LBTD-coupled mass spectrometer was given previously,^29,36^ and we detail here only the parameters pertinent to the current study. A schematic of the setup is shown in Fig. 1. Sample molecules were deposited onto a 10 μm-thick titanium foil (Baoji Energy Titanium Co.) by spraying using a commercially available airbrush gun (Fengda, BD-208, 0.2 mm nozzle, ∼2 bar N_2_), and subsequently drying under air. Thymine (Sigma Aldrich T0376, 99% pure) was applied as an aqueous solution (0.015 M), whilst guanine (Sigma Aldrich G11950, 98% pure), was sprayed from an alkaline (0.05 M NaOH) solution (0.015 M).

Deposited molecules were thermally desorbed into the gas phase by irradiating the uncoated side of the moving titanium foil (125 μm s^−1^) with the continuous output of a diode laser (445 nm, Wavespectrum Laser Inc.) as shown in inset A in Fig. 1. Two knife edges were used to reduce the effective irradiation area on the foil to an approximate size of 3 mm × 0.2 mm (see inset B in Fig. 1). The diode laser power onto the foil (*i.e.*, measured after transmission through the fiber and the knife edges) was set to 0.095 W for thymine and 0.33 W for guanine, below the threshold were desorption-induced fragmentation was observed (ESI†).^29^ The plume of desorbed molecules was ionised by the third (343 nm) or fourth (257 nm) harmonic of an amplified Ytterbium femtosecond laser system with a fundamental output at 1030 nm (Ytterbium-60, Active Fiber Systems GmbH), operated at 50 kHz repetition rate. Typical pulse durations were 250 fs (FWHM). The ionisation laser was focused into the interaction region using a plano-convex spherical lens with a focal length of 500 mm, yielding typical spot sizes of 0.18 mm diameter. The ionisation laser power was varied using a motor-controlled half-wave plate combined with a thin-film polariser. Laser polarisation was kept linear and parallel to the extraction electrodes.

Produced ions were analysed using a custom-built Wiley-McLaren time-of-flight mass spectrometer, operating in ion counting mode and with a typical mass resolution 
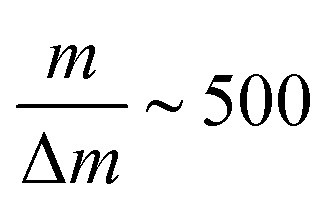
. Single ion hits on the MCP detector were recorded and time-stamped using a combination of constant fraction discriminator (Surface Concept GmbH) and time-to-digital converter (cronologic GmbH). For every laser intensity, the mass spectrum was acquired for ∼50 s collection time, which translates into 60 min for every intensity scan.

## 3. Results and discussion

### 3.1 Thymine

Given the high ionisation energy of thymine, 8.9178 ± 0.01 eV,^37^ its cations were produced through MPI involving overall at least three 343 nm (3.61 eV) or two 257 nm (4.82 eV) photons. In particular, our experimental results show that the ionisation of thymine by 257 nm light is a 1 + 1 resonance-enhanced process through its open-shell singlet state. We discuss this in more detail at the end of this section.

**Fig. 2:**
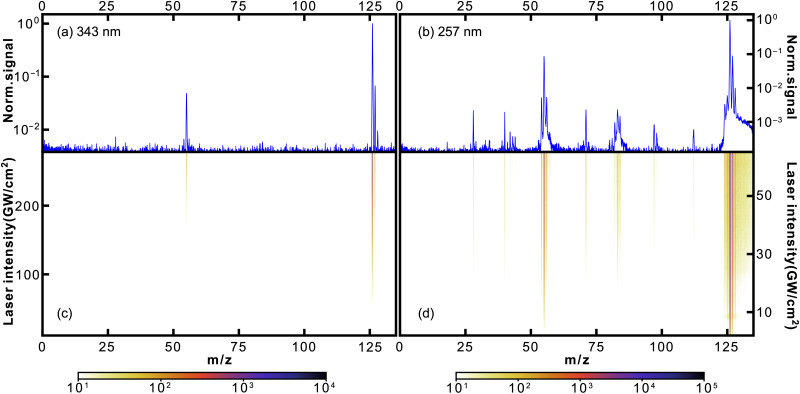
Mass spectra of thymine following 343 nm (a and c) and 257 nm (b and d) fs-MPI. The top panel (a and b) shows the 1D mass spectrum obtained at the highest used intensity, while the 2D heat maps on the bottom (c and d) show the observed mass spectrum as a function of the femtosecond laser intensity. Note the logarithmic intensity scale used throughout.

In Fig. 2, we show the photofragmentation mass spectra of thymine obtained at 343 nm (left) and 257 nm (right) ionisation laser wavelengths. The 2D heat maps (lower panels) show mass spectra obtained as a function of ionisation laser intensity, with the colour scale corresponding to measured ion counts. The upper plots show mass spectra obtained at the highest laser intensities, normalised to the parent ion signal. For both, the logarithmic scale was used.

For both wavelengths, the parent peak had the highest intensity, being at least an order of magnitude larger than the most abundant fragment *m*/*z* = 55. Curiously, this was not the case for the proton-impact fragmentation mass spectrum of thymine obtained by Poully and coauthors^27^ also using LBTD, where the most prominent peak was the *m*/*z* = 55 fragment. This highlights the significance of fs-MPI as a soft ionisation technique. As in previous LBTD studies, we observe no evidence for the formation of clusters.

The rest of the fragment peaks were more than two orders of magnitude smaller than the parent peak. Some of them were resolved quite well in the 257 nm spectrum, while almost all of them being at the noise level in the 343 nm spectrum. Nonetheless, the discussion that follows applies for both wavelengths. In Table 1, we list several of the most abundant fragments resolved in the mass spectra, along with the corresponding yields and appearance intensities. We define the latter as the ionisation laser intensity at which the observed yield reaches 0.5% of the parent ion yield.

**Table 1:** Observed ions following ionisation of thymine using 343 nm or 257 nm femtosecond pulses. Fragment assignments are based on the given literature ref. 1,4,6,8,13 and 15–17. Stated yields are for the mass spectrum corresponding to the highest ionisation laser intensity and relative to the observed parent ion signal. We define the appearance intensity *I*_app_ as the ionisation laser intensity for which the fragment yield reaches 0.5% of parent yield. The exponent *n* is obtained by fitting data with a power law dependence, for details see text and Fig. 4

*m*/*z*	Fragment	343 nm			257 nm		
		Yield (%)	*I*_app_ (GW cm^−2^)	*n*	Yield (%)	*I*_app_ (GW cm^−2^)	*n*
28	CH_2_N^+ ^^4^				0.09		
40	C_3_H_4_N^+^^4^				0.05		
54	C_3_H_4_N^+ ^^4^				0.70	35.5	2.3 ± 0.1
55	C_3_H_5_N^+ ^^1,4,6,8,13,15–17^	8.19	153.1	4.3 ± 0.1	11.96	1.8	2.3 ± 0.1
56	C_3_H_5_N^+^ (C_3_H_3_O^+^)^4^				0.79	28.7	2.3 ± 0.1
71	C_2_HNO_2_^+^				0.19		
83	C_4_H_5_NO^+ ^^4,6^				0.28		
97	C_4_H_3_NO_2_^+ ^^4^				0.03		
124	(Thy-H_2_)^+^				0.58	1.8	2.0 ± 0.1
125	(Thy-H)^+^				1.10	1.8	1.8 ± 0.1
126	Thy^+^	100.00	n/a	3.6 ± 0.1	100.00	n/a	1.4 ± 0.1
127	Thy^+^ ((Thy + H)^+^)	7.97	n/a	4.0 ± 0.1	9.21	n/a	1.6 ± 0.1

**Fig. 3:**
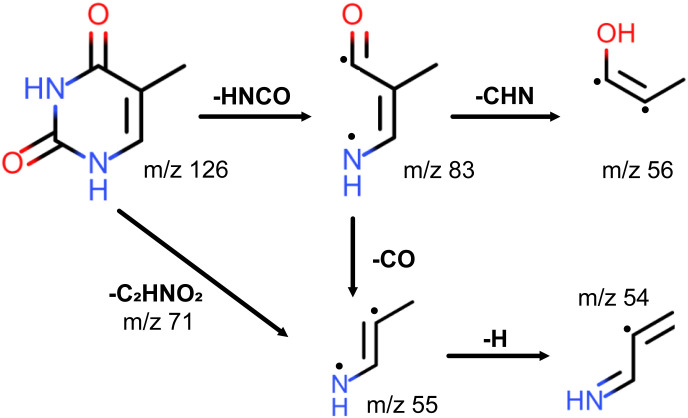
Fragmentation channels of thymine proposed by Jochims and coauthors for *m*/*z* = 83, 56, 55, 54 fragments.^4^ The channels corresponding to HNCO and CO loss were computationally confirmed by Majer and coauthors.^6^ For ionisation at 343 nm only fragment *m*/*z* = 55 is observed.

It is worth noting that the *m*/*z* = 55 fragment was also present as the most prominent peak in previously obtained photofragmentation^4,6^ and electron-impact^1,13^ fragmentation mass spectra of thymine. Furthermore, it was one of the prominent peaks in charged ion or proton impact fragmentation mass spectra.^8,15–17,27^ All of these studies assigned the *m*/*z* = 55 fragment to C_3_H_5_N^+^. In particular, Jochims and coauthors^4^ proposed that C_3_H_5_N^+^ is produced as a result of CO loss from C_4_H_5_NO^+^ after fragmentation of thymine, as shown in Fig. 3. As the main argument they referred to a previous study^38^ which showed that the mass spectrum of thymine, with ^14^C between two nitrogens, retains *m*/*z* = 83 and *m*/*z* = 55 peaks. Their analysis was consistent with the computational results of a previous study by Improta and coauthors.^39^ Recently, the proposed fragmentation pathway has been confirmed and elaborated both experimentally and theoretically by Majer and coauthors.^6^ In addition to confirming HNCO-loss to produce the *m*/*z* = 83 fragment C_4_H_5_NO^+^, which formally corresponds to the retro-Diels–Alder reaction,^40,41^ their calculations showed that CO-loss occurs by formation of a five-membered ring through an internal rotation around the C–CH bond, followed by proton transfer from the methyl group to the nitrogen atom. Even though this CO-loss mechanism yields a particular isomer of C_3_H_5_N^+^, their calculations indicated that three additional low-energy isomers of C_3_H_5_N^+^ exist. The analogous fragmentation pathways were observed for uracil.^42,43^ Alternatively, the *m*/*z* = 55 fragment may be formed directly from the parent ion by losing the *m*/*z* = 71 fragment C_2_HNO_2_ (see Fig. 3),^4^ which was present in the 257 nm mass spectrum.

The *m*/*z* = 127 peak was the third most intense peak in the 257 nm mass spectrum with a yield of 9.2%. The presence of ^13^C only accounts for 5.2% of the *m*/*z* = 127 peak, and we assign the remaining 4% to protonated thymine (Thy + H)^+^.

Considering the prominence of the *m*/*z* = 55 fragment C_3_H_5_N^+^, we assign a considerable portion of the *m*/*z* = 56 peak to C_3_H_5_N^+^ containing one ^13^C. However, the *m*/*z* = 127 thymine parent ion accounts only for about 79% of the *m*/*z* = 56 peak (see Table 1), assuming that the fragment-parent ratio is the same for isotopologues. We assign the rest of it, following Jochims and coauthors,^4^ to C_3_H_4_NO^+^, produced from the *m*/*z* = 83 fragment C_4_H_5_NO^+^ through CHN-loss accompanied with H-transfer to O (see Fig. 3). So, there are two competing pathways for the fragmentation of the *m*/*z* = 83 fragment C_4_H_5_NO^+^, one yielding the *m*/*z* = 55 fragment C_3_H_5_N^+^ and the other the *m*/*z* = 56 fragment C_3_H_4_NO^+^. Recent calculations by Bauer and Grimme^42^ showed that the former is five times more likely than the latter, which is consistent with our experimental results.

We assign the *m*/*z* = 54 peak to the fragment C_3_H_4_N^+^ obtained from the *m*/*z* = 55 fragment through H-loss. The appearance intensity of *m*/*z* = 54 was significantly higher than that of *m*/*z* = 55 (35.5 *vs.* 1.8 GW cm^−2^), which supports the proposed mechanism. Alternatively, *m*/*z* = 54 could be produced from the *m*/*z* = 83 fragment through H-loss followed by CO-loss similar to the mechanism discussed above.

In the 257 nm mass spectrum, besides the peaks discussed, we also resolved several peaks that were almost three orders of magnitude smaller than the parent ion peak, at *m*/*z* = 28, 40, 71, 83, and 97. We have already assigned the *m*/*z* = 71 and *m*/*z* = 83 peaks to C_2_HNO_2_^+^ and C_4_H_5_NO^+^, respectively, when discussing the origin of the *m*/*z* = 55 peak. There is no substantial experimental or computational evidence for the fragmentation mechanisms producing the *m*/*z* = 28, 40, and 97 peaks. For completeness, here we summarize the mechanisms proposed by Jochims and coauthors.^4^ However, it should be noted that they are based on chemical intuition, as pointed out by Majer and coauthors.^6^ The *m*/*z* = 40 peak was assigned to C_3_H_4_N^+^ forming directly from the parent ion through losing two HNCO fragments. The *m*/*z* = 28 peak was assigned to CH_2_N^+^. Jochims and coauthors proposed five different fragmentation channels leading to it. The *m*/*z* = 97 peak was assigned to C_4_H_3_NO_2_^+^ forming directly from the parent ion through CH_3_N loss.

**Fig. 4:**
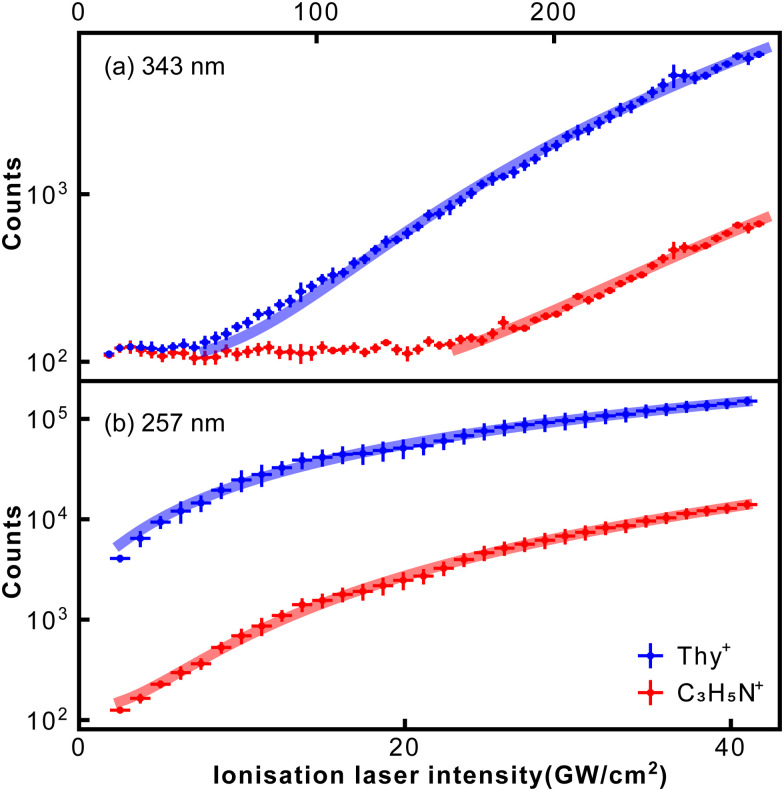
Dependence of thymine parent ion and C_3_H_5_N^+^ fragment signal on the ionisation laser intensity for 343 nm (a) and 257 nm (b). Solid lines correspond to power-law fits.

Fig. 4 shows how abundances of the thymine parent ion and the *m*/*z* = 55 fragment C_3_H_5_N^+^ depend on the ionisation laser intensity for wavelengths of 343 nm (a) and 257 nm (b). The horizontal error bars correspond to bin sizes, whereas the vertical error bars correspond to standard deviations for the data within a bin. The figure also shows corresponding power law fits (*y* = *ax*^*n*^ + *b*). At 257 nm data taken at intensities ≥40 GW cm^−2^ started to show saturation behaviour and was not included in the fitting. The resulting exponents, or photon orders, *n*, are shown in Table 1, which also contains exponents obtained for several other fragments observed using 257 nm fs-MPI.

The exponent obtained for the thymine parent ion at 343 nm was 3.6, which is consistent with the fact that ionisation of thymine requires at least three 343 nm (3.61 eV) photons. Meanwhile, the exponent obtained for the thymine parent ion at 257 nm was 1.4. This value is considerably lower than 2, indicating the formation of thymine cations through a resonance-enhanced, 1 + 1 REMPI, process. These conclusions are backed by the computational results obtained by Fleig and coauthors.^44^ Specifically, they calculated the vertical energies of the three lowest excited electronic states of thymine to be 3.82, 4.61, and 4.82 eV. The last value, which corresponds to the open-shell singlet state of thymine, perfectly matches the energy of 257 nm light. It is worth noting that the transform-limited spectral bandwidth for a 250 fs laser pulse at 343 nm is less than 1 nm, which corresponds to 10 meV. This rules out the REMPI of thymine by 343 nm light through its excited state at 3.82 eV.

The exponent for the *m*/*z* = 55 fragment C_3_H_5_N^+^ was 4.3, which matches the fragment's previously measured appearance energy of ∼12 eV,^4,6^ which requires four 343 nm photons. This appearance energy implies that the fragmentation of *m*/*z* = 55 is supposed to involve at least three 257 nm photons. However, the exponent at 257 nm was 2.3, considerably lower than 3. This indicates that the fragmentation of *m*/*z* = 55 is facilitated by REMPI through the open-shell singlet state of thymine, indicating that fragmentation is driven by absorption of an additional photon after ionisation.^45,46^

In Table 1 we also report the exponents for several other fragments, namely, *m*/*z* = 54, 56, 124, 125. In general, all values were higher than the exponent obtained for thymine parent ion, which is consistent with the fact that fragmentation should involve at least as many photons as ionisation. The exponents for the *m*/*z* = 55 and *m*/*z* = 56 fragments were almost identical, being another confirmation that they both correspond to C_3_H_5_N^+^. Likewise, for the *m*/*z* = 54 we obtained the same exponent as for the *m*/*z* = 55 fragment.

### 3.2 Guanine

Given the known ionisation energy of guanine (IE = 7.8 ± 0.1 eV^47–50^), the MPI process involved at least three 343 nm (3.61 eV) or two 257 nm (4.82 eV) photons. In particular, there was no resonance enhancement as our experimental results showed. We discuss this further at the end of this section, where we analysed the dependence of abundances on the ionisation laser intensity.

**Fig. 5:**
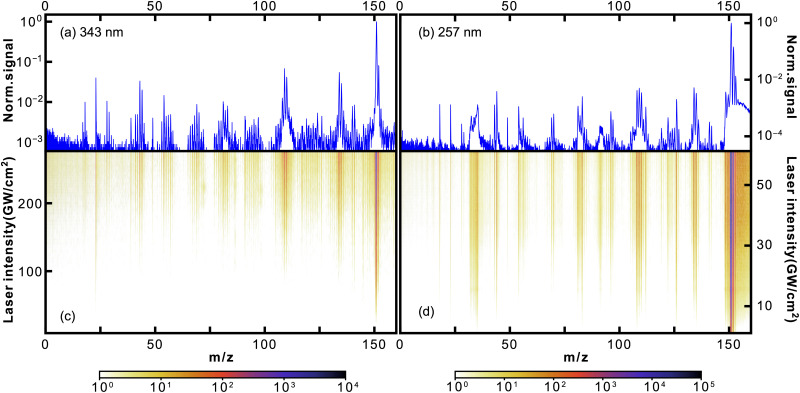
Mass spectra of guanine following 343 nm (a and c) and 257 nm (b and d) fs-MPI. The top panel (a and b) shows the 1D mass spectrum obtained at the highest used intensity, while the 2D heat maps on the bottom (c and d) show the observed mass spectrum as a function of the femtosecond laser intensity. Note the logarithmic intensity scale used throughout.

In Fig. 5, we show the photofragmentation mass spectra of guanine obtained at 343 nm (left) and 257 nm (right) ionisation laser wavelengths. Just as for the thymine mass spectra, the heat maps show mass spectra obtained as a function of ionisation laser intensity, the colours corresponding to ion counts. The plots at the top show mass spectra obtained at the highest laser intensities, normalised to the observed parent ion signal. Note that a logarithmic intensity scale is used for all figures.

Since guanine has a lower ionisation energy and more complex structure than thymine, a much richer fragmentation pattern was observed, as evident from the mass spectra. However, like for thymine, the most abundant peak in all guanine spectra was the parent ion, which was more intense than any fragment peak by at least one order of magnitude for 343 nm, and two orders of magnitude for 257 nm. Given that guanine is prone to thermal decomposition,^9,51^ this strong signal from intact parent ions highlights once more the ‘softness’ of the LBTD molecular source,^29^ as well as the fs-MPI ionisation, with 257 nm light resulting in less fragmentation than 343 nm light. Other than that, the spectra contained almost the same set of peaks. Therefore, the discussion that follows is not specific for a particular wavelength.

The prominent peaks present for both colours were *m*/*z* = 135, 134, 110, 109 and 108. These are well-known fragments of guanine, previously seen in electron-impact fragmentation experiments at electron energies of 60, 70, and 95 eV,^2,9,10,12^ and photofragmentation experiments at photon energies of 16.67 and 21.2 eV.^5^ These fragments, except *m*/*z* = 135, were also produced in collision-induced fragmentation experiments at collision energies from 10 to 50 eV.^20^ One exception is an electron-impact fragmentation study at electron energies of 100 eV that completely missed these fragments, despite having a rich fragmentation mass spectrum.^11^

**Fig. 6:**
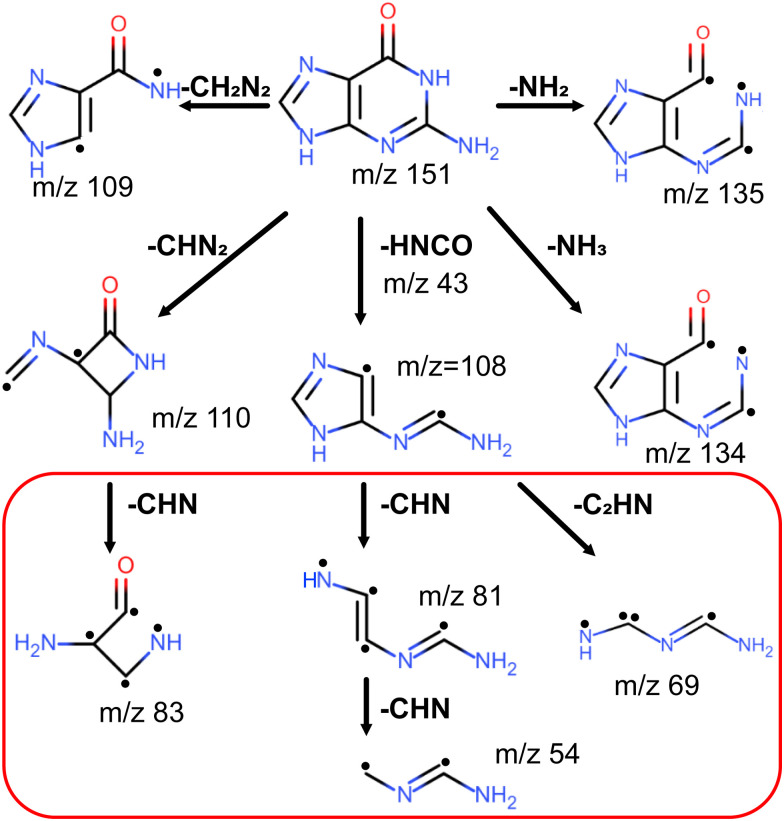
Fragmentation pathways computationally determined by Sard-Arani.^52^ Fragments indicated in the red box are observed in considerable amount for ionisation at 343 nm only.

We assign the peaks *m*/*z* = 135, 134, 110, 109, and 108 to loss of NH_2_, NH_3_, HNCO, CHN_2_, and CH_2_N_2_ fragments from the guanine molecule, respectively, as shown in Fig. 6. These are the same assignments as proposed by Rice and Dudek^2^ in their pioneering work on the fragmentation of guanine. Subsequent experimental studies in general agreed with these assignments, though proposing competing reaction pathways such as *m*/*z* = 135 and 134 forming as a result of O and OH losses from various tautomers of guanine.^5,10,12^ The first serious computational investigation, conducted by Cheng and coauthors^20^ combining DFT and *ab initio* approaches using the B3LYP functional and MP2 method, confirmed Rice and Dudek's assignments, except for *m*/*z* = 135 that was not considered, and proposed final fragment structures along with fragmentation mechanisms. Their results indicated that every fragmentation channel is facilitated by several proton transfer events. The assignments were further confirmed by a computational study conducted by Sadr-Arani and coauthors^52^ at the DFT level using the PBE functional. However, their final structures and fragmentation mechanisms for *m*/*z* = 109 and 110 differed from those obtained by Cheng and coauthors. In particular, for *m*/*z* = 110 the structure by Cheng and coauthors has the intact imidazole ring, whereas the structure by Sadr-Arani and coauthors has both imidazole and pyrimidine rings broken. In Fig. 6, we show structures obtained by Sadr-Arani and coauthors.

We assign the peak at *m*/*z* = 81 to C_3_H_3_N_3_^+^. This peak was present in fragmentation mass spectra of guanine in all aforementioned studies,^2,5,9,10,12,20^ except for one,^11^ and was unanimously assigned to C_3_H_3_N_3_^+^. Moreover, several of the studies^2,9,12^ proposed that the *m*/*z* = 81 fragment C_3_H_3_N_3_^+^ is produced from the *m*/*z* = 109 fragment C_4_H_3_N_3_O^+^ through CO loss. However, Cheng and coauthors^20^ claimed that *m*/*z* = 81 is obtained through HNCO loss from the *m*/*z* = 124 intermediate fragment C_4_H_4_N_4_O^+^, which is obtained from guanine through CHN loss. The calculations by Sadr-Arani and coauthors^52^ produced two pathways: one coinciding with Cheng and coauthors' proposal; and the other reversing the order of CHN and HNCO losses, that is, first producing the *m*/*z* = 108 fragment C_4_H_4_N_4_O^+^ and then the *m*/*z* = 81 fragment C_3_H_3_N_3_^+^. The latter one was also determined to be thermodynamically more efficient. Considering the fact that we observed only a negligible amount of *m*/*z* = 124 in our mass spectra and considerable amounts of *m*/*z* = 108, we favor the latter mechanism.

The situation is more difficult for *m*/*z* = 83. Even though its abundance was comparable to that of *m*/*z* = 81 in our mass spectra, it was present in considerably lower amounts compared to *m*/*z* = 81 in mass spectra of the previous fragmentation studies,^2,9,10,12^ except for the collision-induced fragmentation study by Cheng and coauthors,^20^ where the *m*/*z* = 81 and 83 have almost equal abundances. Unfortunately, Cheng and coauthors did not consider *m*/*z* = 83 in their computational analysis. Nonetheless, Rice and Dudek^2^ proposed that *m*/*z* = 83 is produced from the *m*/*z* = 110 fragment C_4_H_4_N_3_O^+^ through CHN loss. Subsequently two experimental studies^9,12^ supported this proposal. It was also computationally confirmed by Sadr-Arani and coauthors,^52^ whose structure we show in Fig. 6.

The mechanisms for the production of *m*/*z* = 81 and *m*/*z* = 83 from *m*/*z* = 108 and *m*/*z* = 110, respectively, are consistent with the appearance intensities we obtained for these fragments (see Table 2). Specifically, the appearance intensities for *m*/*z* = 108 and *m*/*z* = 110 were lower than those of *m*/*z* = 81 and *m*/*z* = 83, indicating the earlier appearance of *m*/*z* = 108 and *m*/*z* = 110. It is also nicely seen from the heat map for 343 nm in Fig. 5.

**Table 2:** Observed ions following ionisation of guanine using 343 nm or 257 nm femtosecond pulses. Fragment assignments are based on the given literature ref. 2,5,52. Stated yields are for the mass spectrum obtained at the highest ionisation laser intensity and relative to the observed parent ion signal. We define the appearance intensity *I*_app_ as the ionisation laser intensity for which the fragment yield reaches 0.5% of parent yield. The exponent *n* is obtained by fitting data with a power law dependence, for details see text and Fig. 7

*m*/*z*	Fragment^2,52^	343 nm			257 nm		
		Yield (%)	*I*_app_ (GW cm^−2^)	*n*	Yield (%)	*I*_app_ (GW cm^−2^)	*n*
28	CH_2_N^+ ^^5^	1.23	123.4	6.4 ± 0.3	0.02		
43	CH_3_N_2_^+^	3.61	84.0	5.4 ± 0.1	0.11		
44	CH_4_N_2_^+^	2.82	87.6	5.5 ± 0.3	0.38		2.8 ± 0.1
54	C_2_H_2_N_2_^+^	2.29	103.9	5.7 ± 0.1	0.12		
69	C_2_H_3_N_3_^+^	1.22	91.4	4.0 ± 0.1	0.05		
81	C_3_H_3_N_3_^+^	1.67	87.6	4.2 ± 0.1	0.14		
83	C_3_H_3_N_2_O^+^	1.19	91.4	4.0 ± 0.1	0.26		
108	C_4_H_4_N_4_O^+^	2.25	80.5	4.0 ± 0.1	0.46		2.2 ± 0.1
109	C_4_H_3_N_3_O^+^	8.31	63.8	4.1 ± 0.1	0.50	39.6	2.9 ± 0.1
110	C_4_H_4_N_3_O^+^	5.86	70.9	4.0 ± 0.1	0.34		2.9 ± 0.1
134	C_5_H_2_N_4_O^+^	6.80	65.1	4.0 ± 0.1	0.44		2.9 ± 0.1
135	C_5_H_3_N_4_O^+^	3.65	70.9	4.1 ± 0.1	0.32		2.3 ± 0.1
151	Gua^+^	100.00	n/a	3.1 ± 0.1	100.00	n/a	1.8 ± 0.1
152	Gua^+^ ((Gua + H)^+^)	8.75	n/a	3.0 ± 0.1	12.20	n/a	2.1 ± 0.1

We assign the peak *m*/*z* = 69 to C_2_H_3_N_3_^+^. This peak was the most abundant one in its vicinity in fragmentation mass spectra of almost all previous studies.^2,5,9,10,12^ This was also the case for our 343 nm spectrum. However, in our 257 nm spectrum it was slightly less abundant than the *m*/*z* = 70 peak. All previous studies that considered the *m*/*z* = 69 peak, assigned it to C_2_H_3_N_3_^+^.^5,9,10,12,20^ Cheng and coauthors proposed that it forms from *m*/*z* = 124, first losing CO and forming an intermediate *m*/*z* = 96, then losing CHN. Even though Sadr-Arani and coauthors computationally confirmed the possibility of this channel, their calculations also indicated an alternative channel through C_2_HN loss from the *m*/*z* = 108 fragment C_4_H_4_N_4_O^+^. As seen from Fig. 6, this channel competes with *m*/*z* = 81 formation from *m*/*z* = 108, though its formation involves breaking a different bond in the imidazole ring according to calculations by Sadr-Arani and coauthors. Considering the abundances of *m*/*z* = 108 and *m*/*z* = 124, we prefer the latter mechanism.

We assign the peak *m*/*z* = 54 to C_2_H_2_N_2_^+^, in agreement with all the previous studies.^2,5,9,10,12,20,52^ Both experimental and theoretical studies concluded that the *m*/*z* = 54 fragment is formed from the *m*/*z* = 81 fragment C_3_H_3_N_3_^+^ through CHN loss, though the calculations by Sadr-Arani and coauthors indicated the possibility of its formation directly from the *m*/*z* = 108 fragment without forming the *m*/*z* = 81 intermediate.

The situation is more uncertain with the *m*/*z* = 43 and 44 peaks. The *m*/*z* = 43 peak was present as the most abundant fragment in all previously obtained electron-impact fragmentation mass spectra.^2,5,9,10,12^ The same is true for photofragmentation spectra obtained at 16.67 eV and 21.2 eV photon energies.^5^ The *m*/*z* = 44 peak was also present but in smaller amounts, with the exception of 16.67 eV photofragmentation spectrum where the *m*/*z* = 44 was as intense as *m*/*z* = 43. In contrast, in this work the peaks *m*/*z* = 43 and 44 were not the most prominent, giving that role to larger fragments *m*/*z* = 108, 109, 110, 134, and 135. This fact too highlights the softness of our fragmentation experiment. These peaks were not present in collision-induced fragmentation spectra obtained by Cheng and coauthors.^20^

Rice and Dudek^2^ assigned the *m*/*z* = 43 and 44 peaks to CH_3_N_2_^+^ and CH_4_N_2_^+^. The subsequent studies followed suit in addition to suggesting CHNO^+^ and CH_2_NO^+^ as possibilities.^5,9,10^ The calculations by Sadr-Arani and coauthors indicated several fragmentation channels leading to *m*/*z* = 43 and 44, and all of them resulted in the CH_3_N_2_^+^ and CH_4_N_2_^+^ fragments. Hence, we assign the *m*/*z* = 43 and 44 peaks to CH_3_N_2_^+^ and CH_4_N_2_^+^.

The *m*/*z* = 28 peak is assigned to CH_2_N^+^, as is done by others.^5,10,12^ Plekan and coauthors^5^ also noted the possibility of it being CO, which we consider unlikely considering that CO has a high ionisation energy (14.01 eV^53^) and would likely not be ionised by the laser intensities used here. Sadr-Arani and coauthors^52^ obtained multiple pathways all resulting in CH_2_N^+^.

**Fig. 7:**
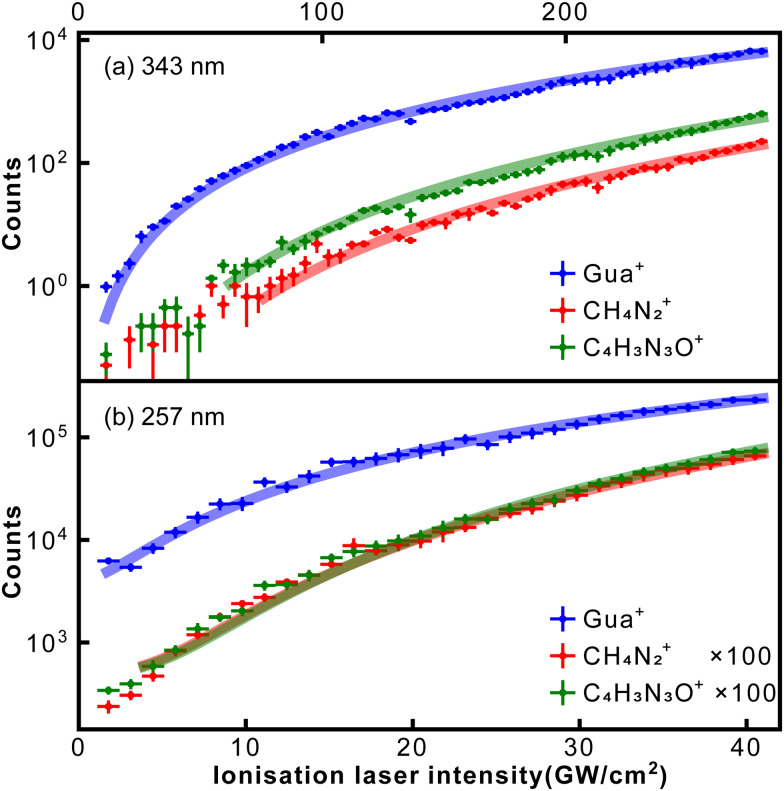
Dependence of guanine parent, CH_4_N_2_^+^ and C_4_H_3_N_3_O^+^ fragments signal on ionisation laser intensity. The ionisation laser wavelength for (a) is 343 nm and for (b) is 257 nm. In (b) the fragment intensities are multiplied by 100. Solid lines correspond to power law fits, see text for details.

In Fig. 7, we show the measured signal of guanine parent ion and its two most abundant fragments as a function of ionisation laser intensity for both wavelengths, along with power law fits for each species. As for thymine, data taken with the highest 257 nm intensities started to show saturation behaviour and was not included in the fitting. The same power law analysis was also performed for all the main fragments listed in Table 2, which contains the resulting exponents.

For 343 nm the exponents for both isotopologues of guanine were almost 3, which is consistent with the fact that the ionisation of guanine requires three 343 nm photons. Likewise, for 257 nm the exponents were 1.8 and 2.1, matching the ionisation of guanine by two 257 nm photons. In particular, these results indicate the absence of resonance enhancement. Furthermore, Fleig and coauthors^44^ calculated the energies of the three lowest excited states of guanine to be 4.29, 4.41, and 4.98 eV. Considering that the transform-limited spectral bandwidth of our 250 fs laser pulse at 257 nm was less than 0.5 nm, none of these states is going to facilitate REMPI.

For all the fragment ions we obtained larger exponents. Specifically, for 343 nm the exponents for larger fragments (*m*/*z* ≥ 69) were all around 4, whereas for smaller fragments (*m*/*z* ≤ 54) the exponents were between 5.4 and 6.4. For 257 nm, the exponents for most of the fragments were around 3. In both cases confirming the absorption of additional photons after ionisation.^45,46^

## 4. Conclusion

We combined laser-based thermal desorption and femtosecond multiphoton ionisation to perform high-throughput fragmentation studies of thymine and guanine. Specifically, the fragmentation spectra of thymine and guanine at 257 nm and 343 nm were reported for the first time. For both thymine and guanine the fs-MPI method yielded a very soft ionisation with the parent ion signal being at least an order of magnitude larger than any observed fragment signal.

Observed fragments for both thymine and guanine, along with the corresponding fragmentation channels, were discussed in comparison with previous experimental and theoretical studies. In the case of thymine a single major fragmentation channel dominated, namely, the one producing the *m*/*z* = 55 fragment C_3_H_5_N^+^ through consecutive HNCO and CO losses. In contrast, guanine showed a rich fragmentation pattern involving several competing channels, with the first step in almost all considered fragmentation mechanisms being the opening of the pyrimidine ring.

The high repetition rate of our experiment allowed us to analyse the intensity dependence of the observed fragmentation mass spectra in great detail. This confirmed the photon order of the observed ionisation processes, with guanine being non-resonantly ionised by two photons at 257 nm or three photons at 343 nm, and we report the first appearance intensities and photon orders for multiphoton ionisation/fragmentation of guanine. For thymine ionisation at 343 nm was also non resonant (three photons), while a clear resonance enhancement was seen for 257 nm ionisation. Furthermore, the detailed 2D ‘heat maps’ can be considered a unique fingerprint of a particular molecular structure and can help unravel complex fragmentation pathways.

## Conflicts of interest

There are no conflicts to declare.

## Supplementary Material

CP-025-D3CP00328K-s001

## References

[cit1] Rice J. M., Dudek G. O., Barber M. (1965). J. Am. Chem. Soc..

[cit2] Rice J. M., Dudek G. O. (1967). J. Am. Chem. Soc..

[cit3] Watson J., Crick F. (1953). Nature.

[cit4] Jochims H.-W., Schwell M., Baumgartel H., Leach S. (2005). Chem. Phys..

[cit5] Plekan O., Feyer V., Richter R., Coreno M., de Simone M., Prince K. C. (2007). Chem. Phys..

[cit6] Majer K., Signorell R., Heringa M. F., Goldmann M., Hemberger P., Bodi A. (2019). Chem. – Eur. J..

[cit7] Schwell M., Jochims H.-W., Baumgärtel H., Dulieu F., Leach S. (2006). Planet. Space Sci..

[cit8] Imhoff M., Deng Z., Huels M. A. (2005). Int. J. Mass Spectrom..

[cit9] Zavilopulo A. N., Shpenik O. B., Agafonova A. S. (2009). J. Phys. B.

[cit10] Minaev B. F., Shafranyosh M. I., Svida Y. Y., Sukhoviya M. I., Shafranyosh I. I., Baryshnikov G. V., Minaeva V. A. (2014). J. Chem. Phys..

[cit11] Rahman M. A., Krishnakumar E. (2016). J. Phys. Chem..

[cit12] Ostroverkh A., Zavilopulo A., Shpenik O. (2019). Eur. Phys. J. D.

[cit13] van der Burgt P. J., Mahon F., Barrett G., Gradziel M. L. (2014). Eur. Phys. J. D.

[cit14] Dawley M. M., Tanzer K., Cantrell W. A., Plattner P., Brinkmann N. R., Scheier P., Denifl S., Ptasińska S. (2014). Phys. Chem. Chem. Phys..

[cit15] Padellec A. L., Moretto-Capelle P., Richard-Viard M., Champeaux J. P., Cafarelli P. (2008). J. Phys.: Conf. Ser..

[cit16] Tabet J., Eden S., Feil S., Abdoul-Carime H., Farizon B., Farizon M., Ouaskit S., Märk T. D. (2010). Int. J. Mass Spectrom..

[cit17] Vries J. d, Hoekstra R., Morgenstern R., Schlathölter T. (2004). Phys. Scr..

[cit18] Brédy R., Bernard J., Chen L., Montagne G., Li B., Martin S. (2009). J. Chem. Phys..

[cit19] de Vries J., Hoekstra R., Morgenstern R., Schlathölter T. (2003). Phys. Rev. Lett..

[cit20] Cheng P., Li Y., Li S., Zhang M., Zhou Z. (2010). Phys. Chem. Chem. Phys..

[cit21] Ciażyńska M., Olejniczak-Staruch I., Sobolewska-Sztychny D., Narbutt J., Skibińska M., Lesiak A. (2021). Life.

[cit22] Alizadeh E., Orlando T. M., Sanche L. (2015). Annu. Rev. Phys. Chem..

[cit23] Gao Y., Zheng Y., Sanche L. (2021). Int. J. Mol. Sci..

[cit24] Xie J., Gong L., Zhu S., Yong Y., Gu Z., Zhao Y. (2019). Adv. Mater..

[cit25] Lindner B., Seydel U. (1985). Anal. Chem..

[cit26] Golovlev V. V., Allman S. L., Garrett W. R., Taranenko N. I., Chen C. H. (1997). Int. J. Mass Spectrom. Ion Processes.

[cit27] Poully J.-C., Miles J., De Camillis S., Cassimi A., Greenwood J. B. (2015). Phys. Chem. Chem. Phys..

[cit28] Bocková J., Rebelo A., Ryszka M., Pandey R., Mészáros D., Limão-Vieira P., Papp P., Mason N. J., Townsend D., Nixon K. L., Vizcaino V., Poully J.-C., Eden S. (2021). RCS Adv..

[cit29] Wang S., Abma G. L., Krüger P., van Roij A., Balster M., Janssen N., Horke D. A. (2022). Eur. Phys. J. D.

[cit30] Sparling C., Crane S. W., Ireland L., Anderson R., Ghafur O., Greenwood J. B., Townsend D. (2023). Phys. Chem. Chem. Phys..

[cit31] Tanaka K., Waki H., Ido Y., Akita S., Yoshida Y., Yoshida T., Matsuo T. (1988). Rapid Commun. Mass Spectrom..

[cit32] Fenn J. B., Mann M., Meng C. K., Wong S. F., Whitehouse C. M. (1989). Science.

[cit33] Takáts Z., Wiseman J. M., Gologan B., Cooks R. G. (2004). Science.

[cit34] de Vries M. S., Hobza P. (2007). Annu. Rev. Phys. Chem..

[cit35] Teschmit N., Długołecki K., Gusa D., Rubinsky I., Horke D. A., Küpper J. (2017). J. Chem. Phys..

[cit36] Huang Z., Ossenbrüggen T., Rubinsky I., Schust M., Horke D. A., Küpper J. (2018). Anal. Chem..

[cit37] Choi K.-W., Lee J.-H., Kim S. K. (2005). J. Am. Chem. Soc..

[cit38] Ulrich J., Teoule R., Massot R., Cornu A. (1969). Org. Mass Spectrom.

[cit39] Improta R., Scalmani G., Barone V. (2000). Int. J. Mass Spectrom..

[cit40] Dougherty R. C. (1968). J. Am. Chem. Soc..

[cit41] Budzikiewicz H., Brauman J. I., Djerassi C. (1965). Tetrahedron.

[cit42] Bauer C. A., Grimme S. (2015). Eur. J. Mass Spectrom..

[cit43] Pandey R., Ryszka M., da Fonseca Cunha T., Lalande M., Dampc M., Limão-Vieira P., Mason N. J., Poully J. C., Eden S. (2017). Chem. Phys. Lett..

[cit44] Fleig T., Knecht S., Hättig C. (2007). J. Phys. Chem. A.

[cit45] Gunzer F., Krüger S., Grotemeyer J. (2019). Mass Spectrom. Rev..

[cit46] Grotemeyer J., Schlag E. W. (1988). Angew. Chem., Int. Ed. Engl..

[cit47] Orlov V., Smirnov A., Varshavsky Y. (1976). Tetrahedron Lett..

[cit48] Dougherty D., Younathan E., Voll R., Abdulnur S., McGlynn S. (1978). J. Electron Spectrosc. Relat. Phenom..

[cit49] Verkin B., Sukodub L., Yanson I. (1976). Doklady Akad. Nauk SSSR.

[cit50] Hush N. S., Cheung A. S. (1975). Chem. Phys. Lett..

[cit51] Nir E., Plützer C., Kleinermanns K., de Vries M. (2002). Eur. Phys. J. D.

[cit52] Sadr-Arani L., Mignon P., Chermette H., Abdoul-Carime H., Farizon B., Farizon M. (2015). Phys. Chem. Chem. Phys..

[cit53] Erman P., Karawajczyk A., Rachlew-Källne E., Strömholm C., Larsson J., Persson A., Zerne R. (1993). Chem. Phys. Lett..

