# Supplementary material for: High-throughput UV-photofragmentation studies of thymine and guanine
Source: Phys Chem Chem Phys. 2023 Apr 17;25(17):12322–30. doi: 10.1039/d3cp00328k (PMC10155487; doi:10.1039/d3cp00328k)
Supplement: CP-025-D3CP00328K-s001 [file CP-025-D3CP00328K-s001.pdf]

Electronic Supplementary Material (ESI) for Physical Chemistry Chemical Physics.
This journal is © the Owner Societies 2023

Supplementary Information: High-throughput UV-photofragmentation studies of thymine and guanine

Siwen Wang,[1] Yerbolat Dauletyarov,[1] Peter Krüger,[1] and Daniel A. Horke[1,*]

[1] *Radboud University, Institute for Molecules and Materials, Heyendaalseweg 135, 6525 AJ Nijmegen, The Netherlands*

(Dated: March 30, 2023)

* d.horke@science.ru.nl

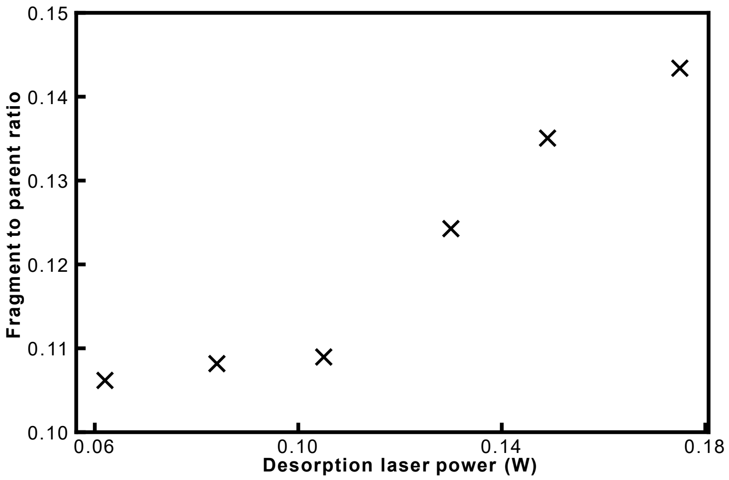

FIG. S1. Fragment-to-parent ratio as a function of desorption laser power for the $C_3H_5N^+$ fragment of thymine.

Desorption-power dependence

Desorption laser powers used for thymine and guanine were optimised to maximise the observed ion signal, while ruling out any fragmentation induced by the desorption laser. To achive this, the desorption power is scanned for a fixed (high) ionisation laser intensity, and the resulting fragment-to-parent ratio monitored. With increasing desorption power this typically shows a flat plateau until some critical power, after which it increased steeply indicating that fragmentation is induced by the desorption process [1]. Experiments are then performed at desorption laser powers below this critical value.

In Figure S1 we show the observed fragment-to-parent ratio of thymine (for the $C_3H_5N^+$ fragment) as a function of desorption laser intensity. A clear plateau is observed until around 0.11 W, followed by a sharp increase afterwards. The experiments presented in the main manuscript were performed at desorption laser powers of 0.095 W.

In Figure S2 we show the observed fragment-to-parent ratio of guanine (for the $C_5H_3N_4O^+$ fragment) as a function of desorption laser intensity. A clear plateau is observed, with only a slight increase at the highest desorption laser power of 0.5 W (the limit of our laser diode). The experiments presented in the main manuscript were performed at desorption laser powers of 0.33 W.

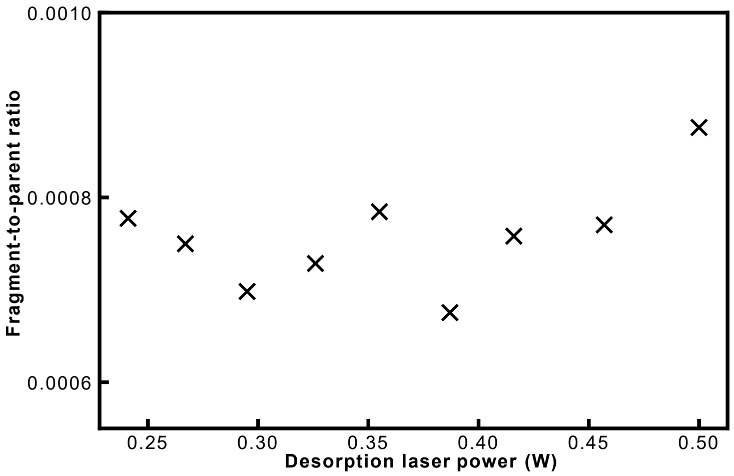

FIG. S2. Fragment-to-parent ratio as a function of desorption laser power for the $C_5H_3N_4O^+$ fragment of guanine.

References

[1] S. Wang, G. L. Abma, P. Krüger, A. van Roij, M. Balster, N. Janssen and D. A. Horke, *Eur. Phys. J. D*, 2022, **76**, 128.
